# New insights into two distinct nucleosome distributions: comparison of cross-platform positioning datasets in the yeast genome

**DOI:** 10.1186/1471-2164-11-33

**Published:** 2010-01-15

**Authors:** Jihua Feng, Xianhua Dai, Qian Xiang, Zhiming Dai, Jiang Wang, Yangyang Deng, Caisheng He

**Affiliations:** 1 School of Information Science and Technology, Sun Yat-Sen University, 135 West Xin'gang Road, Guangzhou, PR China

## Abstract

**Background:**

Recently, a number of high-resolution genome-wide maps of nucleosome locations in S. cerevisiae have been derived experimentally. However, nucleosome positions are determined in vivo by the combined effects of numerous factors. Consequently, nucleosomes are not simple static units, which may explain the discrepancies in reported nucleosome positions as measured by different experiments. In order to more accurately depict the genome-wide nucleosome distribution, we integrated multiple nucleosomal positioning datasets using a multi-angle analysis strategy.

**Results:**

To evaluate the contribution of chromatin structure to transcription, we used the vast amount of available nucleosome analyzed data. Analysis of this data allowed for the comprehensive identification of the connections between promoter nucleosome positioning patterns and various transcription-dependent properties. Further, we characterised the function of nucleosome destabilisation in the context of transcription regulation. Our results indicate that genes with similar nucleosome occupancy patterns share general transcription attributes. We identified the local regulatory correlation (LRC) regions for two distinct types of nucleosomes and we assessed their regulatory properties. We also estimated the nucleosome reproducibility and measurement accuracy for high-confidence transcripts. We found that by maintaining a distance of ~13 bp between the upstream border of the +1 nucleosome and the transcription start sites (TSSs), the stable +1 nucleosome may form a barrier against the accessibility of the TSS and shape an optimum chromatin conformation for gene regulation. An in-depth analysis of nucleosome positioning in normally growing and heat shock cells suggested that the extent and patterns of nucleosome sliding are associated with gene activation.

**Conclusions:**

Our results, which combine different types of data, suggest that cross-platform information, including discrepancy and consistency, reflects the mechanisms of nucleosome packaging in vivo more faithfully than individual studies. Furthermore, nucleosomes can be divided into two classes according to their stable and dynamic characteristics. We found that two different nucleosome-positioning characteristics may significantly impact transcription programs. Besides, some positioned-nucleosomes are involved in the transition from stable state to dynamic state in response to abrupt environmental changes.

## Background

In eukaryotic organisms, the association of DNA with histone octamers to form repeating nucleosome units has profound implications for all aspects of cellular metabolism. In particular, the histone components, as well as additional chromatin proteins, can interact to form higher order chromosomal structures. Thus, nucleosomes are critical to the organisation and maintenance of chromatin, and their position and modification state can significantly influence genetic activities, such as the plasticity or control of gene expression. As a result, studies of nucleosome positions, determined by either experimental measurements or computational methods, continue to be an active field of research [1-11].

Six high-resolution genome-scale nucleosome positioning studies have recently been completed in S. cerevisiae [2-6,11]. In these assays, either tiling arrays or direct sequencing technologies were used to map the positions of nucleosomes. However, it is clear from previous work that nucleosome positions are subtle and diffuse, which makes it difficult to distinguish their true position data from biological noise in a single experiment [1-8]. The biological dynamics under different experimental conditions that may be responsible for inconsistencies among these studies led us to develop a criterion to assess these studies effectively. In addition, inconsistent assignment of nucleosome positions, derived from different detection methods, highlights the need for careful and comprehensive comparison of these experimental datasets.

Here, we overcame the limitations of single study analyses by pooling the nucleosome distribution information from six independent datasets [1-5,11] so that valid relationships were reinforced and biological noise was suppressed. Through the use of multi-angle probing of the cross-platform datasets, whether under the same or different conditions, we sought to address the following problems: (1) What are the points of agreement and the disagreement between these cross-platform experimental datasets? (2) Can this cross-platform information reflect the mechanism of nucleosome packaging in vivo more faithfully than an individual study? (3) What are the relationships between the two classes of nucleosome positioning patterns and regulatory properties, such as transcription rate [12], mRNA abundance [13], sensitivity to chromatin regulation [14], and histone turnover [15]? (4) How do cells use both random deposition and specific positioning of nucleosomes to connect with gene architecture, such as TATA-containing and TATA-free promoters [16]?

## Results

### Comparisons among cross-platform nucleosomal datasets in different genome regions

To objectively compare published nucleosome position data, we first collected all available basic information from these studies. All six experiments measured genome-wide nucleosome positions, but differed in their focus, emphasis and platforms (Table 1). We divided these experiments into two groups according to the strains used and the experimental conditions. The normal group was defined as those studies that primarily made nucleosome preparations from BY4741 strains under normal conditions: Lee et al. [3], Albert et al. [2], Mavrich et al. [5] and Field et al[11]. The conditional group consisted of the studies that used S288C strains and conducted experiments in the context of a physiological or genetic perturbation: Whitehouse et al. [4] and Shivaswamy et al[6].

**Table 1:** Summary of platforms

Authors	Strains/Culture	Platform	Detection strategy	Number/Resolution
Lee et al.	BY4741/in YPD	Affymetrix	HMM	70,871/4 bp
Albert et al.	BY4741/in rich media	Pyrosequencing	Chip-Seq Length:~25 bp	~10,000/~4 bp
Whitehouse et al.	S288C/in C media	Affymetrix	Iteratively fitting	*WT*:58,275/~5 bp
				*M*:62,594/~5 bp
Shivaswamy et al.	S288C/in rich media	Solexa Ultra-high-throughput sequencing	Chip-Seq	*N*:49,043/~1 bp
			Length:~25 bp	*H*:52,817/~1 bp
Mavrich et al.	BY4741/YPD	The Roche GS20	Chip-Seq	54,753/~1 bp
		454 Life Sciences	Length > 100 bp	
Field et al.	BY4741/in YPD	454 pyrosequencing	Chip-Seq	43,720~44,134/~1 bp
		parallel sequencing	Length:~200 bp	

The normal condition is denoted by N, and the heat shock condition is denoted by H. The isw2 mutation strains and wild-type strains are separately represented by M and WT. Numbers in the fifth column represent the total nucleosome counts detected in each experiment and data resolution, respectively.

In order to roughly evaluate the discrepancies and consistency between the six datasets, we calculated the Pearson correlation coefficients between the nucleosome positioning maps in different genomic regions.

**Figure 1:**
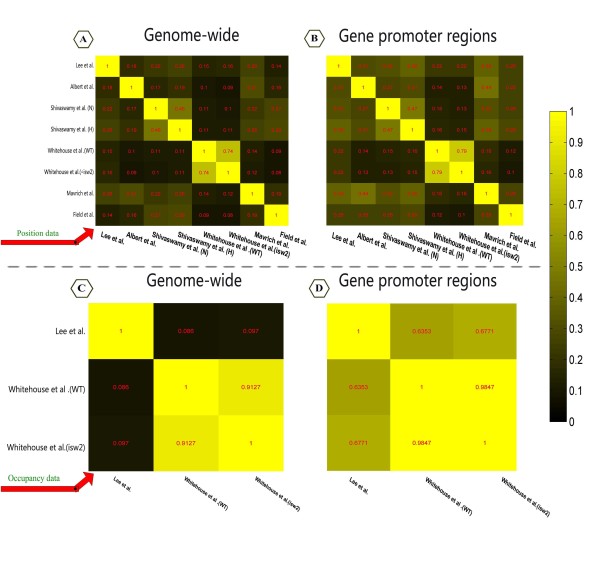
**The correlation coefficient between cross-platform nucleosome positioning datasets**. In the heat maps, the red number represents corresponding correlation coefficients between datasets. (A),(B) The correlation coefficients matrix of the six position datasets are plotted as heat maps [2-6,11], and (C), (D) The correlation coefficients matrix of the three occupation ratio datasets are plotted as heat maps[3,4]. (A) and (C) represent genome-wide correlations between datasets, whereas (B) and (D) represent promoter region correlations between datasets.

**Figure 2:**
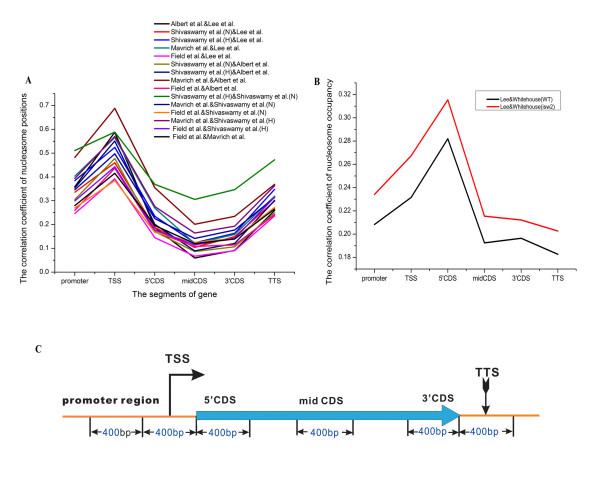
**The correlation coefficients of nucleosome position datasets in the different gene segments**. (A) The average correlation coefficients of the six position datasets [2-6,11] in the different regions of genes. (B) The average correlation coefficients of the two occupation ratio datasets [3,4] in different gene regions.(C) Nucleosome occupancy on a gene is separated into the following five regions: promoter, TSS, 5'CDS, mid CDS, 3'CDS and TTS. Every region spans 400 bp.

• According to positioning data [2-6,11], the average correlation coefficient is 0.21 across the entire genome and 0.29 in promoter regions (Figure 1A, B). According to the occupancy ratio data [3,4], the average correlation coefficient is 0.1 across the entire genome and 0.65 in promoter regions (Figure 1C, D).

• In the analysis of different gene segments, the most consistent regions for nucleosome positions are the TSSs, according to positioning data. Intriguingly, according to the occupancy ratio data, the most consistent region for nucleosome positions is the 5' end of Coding DNA Sequences (5' CDSs) (Figure 2A, B).

Notably, we found that the correlations between the Whitehouse et al. study and the others are significantly lower than the average, regardless of the regions and data type (Figure 1). To explore this discrepancy further, we learned that both the Lee et al. and Whitehouse et al. studies used the same experimental platform, but that they differed in the strains used, data normalization methods and in their associated position detecting methods (Table 1). For nucleosome position detection, Lee et al. used the popular hidden Markov model (HMM) to obtain final nucleosome positions [3], similar to the method employed by Yuan et al. [1]. In contrast, Whitehouse et al. determined nucleosome positions by iteratively fitting an idealised nucleosome signal to the occupancy ratio data [4].

Besides position detecting methods, a potential artifact in the nucleosome mapping experiments is micrococcal nuclease (MNase) used, which might also lead to difference between those nucleosome positioning datasets. While this enzyme is universally used to isolate nucleosome core DNA by preferentially digesting linker DNA to release the mononucleosome cores, its activity is not without sequence biases [17-19]. Consequently, these sequencing biases are usually corrected by normalization with control sequences. However, we noted that another difference between the data from Whitehouse et al. and Lee et al. is that the former compared DNase treated nucleosomal DNA to nucleosomal DNA that had not been treated with DNase, while Lee et al. normalized against DNase treated genomic DNA. Thus, this difference should influence not only on the analyzed (nucleosome positioning) data but also on the raw (nucleosome occupancy) data.

**Figure 3:**
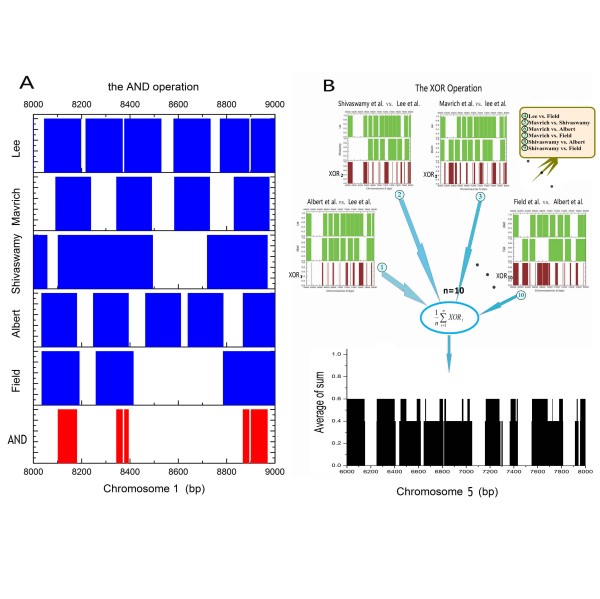
**Data restructuring and processing**. The demonstration of five datasets [2,3,5,6,11] (under normal conditions) processing in two arbitrary chromosome segments. Packaging DNA is represented by the logical symbol 1 and linker DNA is represented by 0 along the chromosome coordinates. (A) The logical AND operation procedure (chromosome1: 8000~9000 bp). (B) The logical XOR operation procedure (chromosome5: 6000~8000 bp): All 10 pairwise combinations of five datasets were calculated using the XOR operator. Finally, the arithmetic mean value was calculated from these XOR results.

In order to resolve the disagreement between datasets, we used binary sequences to reconstruct the nucleosome position datasets. Under this method, packaging DNA is represented by the logical symbol 1 and linker DNA is represented by 0 along the chromosome coordinates (Figure 3). All the new binary position datasets were aligned and averaged with respect to the TSS (see Methods or Additional file 1: Supplemental Figure S1). As expected, we found that the position pattern of the Whitehouse et al. data is weaker than the pattern observed in the other datasets (data not shown). However, when analysing promoter regions, we noted that the occupancy ratio data from the Whitehouse et al. study suggests that the same features are significant as identified in other studies (data not shown). Several factors might contribute to this, including noise of probe hybridisation, MNase bias correction, and the specific methods used for peak calling/detecting. We speculate that the significant discrepancies between the positioning data of Whitehouse et al. and the other studies are largely derived from the methods used for final nucleosome position detection. Furthermore, the data from Albert et al. also shows only moderate correlation with other datasets. However, the H2A.Z nucleosomes detected in their study are only a subset of all cellular nucleosomes, accounting for ~20% of the total [2,20,21].

It is important to point out that to date there exist only two high-resolution genome-wide occupancy ratio datasets derived from microarrays [3,4]. Therefore, in a statistical sense, the analysis of nucleosomal occupancy data (i.e., raw data) is more uncertain than that of positional data (i.e., analyzed data). Based on the previously discussed factors, our study was restricted to the six recent nucleosomal positioning datasets, including five datasets [2,3,5,6,11] under normal conditions and one [6] under a stress condition.

### Two distinct nucleosome positioning patterns

To decipher nucleosome positioning patterns from the cross-platform datasets, it is vital to determine the agreements and disagreements between these datasets. In a computationally intensive analysis, we identified these relationships by using the conjunction operation (logic AND) and the exclusive or operation (logic XOR) (see Methods). We chose these two methods for the following reasons: (1) The logical method is simple, rapid and accurate, which is very suitable for our restructuring binary data. (2) The logical algorithm has a good performance in large binary datasets: its computational cost is smaller than that of the real.

**Figure 4:**
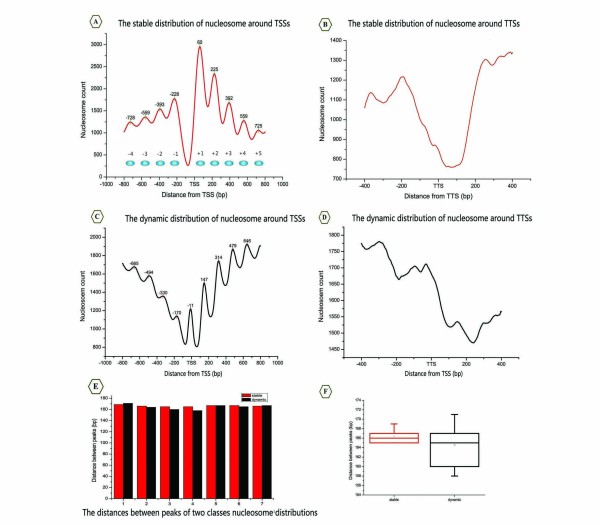
**Two distinct distribution domains around the TSS and the TTS**. (A) The stable nucleosome distribution profile around the TSS. The digital values represent the coordinates of peaks relative to the TSS. (B) The stable nucleosome distribution profile around the TTS. (C) The dynamic nucleosome distribution profile around the TSS. The data represent the locations of peaks from the TSS. (D) The dynamic nucleosome distribution profile around the TTS. (E) The distances between peaks. Red bars represent the span of stable nucleosome profile peaks, whereas black bars indicate the span of dynamic nucleosome profile peaks. (F) Box plot of average peak distances. The red box represents the stable nucleosome, while the black box represents the dynamic nucleosome.

First, we performed a logical AND to extract common information from the six binary datasets (Figure 3), which include one heat shock dataset [6] and five normal condition datasets [2,3,5,6,11]. In principle, the intersection of the six binary datasets represents the stable nucleosome distribution among these datasets. Indeed, when we aligned and averaged the intersection signals with the TSS, an orderly organisational profile emerged from the promoter regions. Unlike the results of previous studies [3,5,6], however, we observed a natural order decay of stable nucleosome positioning peaks in the flanks of the nucleosome-free regions (NFRs) that depends on the distance from the TSSs. Despite the fact that our data were gathered from different platforms and under various conditions, all of the common combinatorial signals exhibit uniform phases and are distributed symmetrically around the TSS, both findings that have not been previously reported (Figure 4A). We referred to these in-phase signals as the stable nucleosome profile. This finding suggests that nucleosome organisation in the genome may be more conserved than previously thought. As shown in Figure 4A, these peaks of distribution profiles were referred to as "stable equilibrium points", which reflect the expected values of nucleosome centres according to probability theory.

The observed discrepancies suggested that dynamic characteristics are inherent to the nucleosomes. To begin with, the inconsistent data are defined as follows. If and only if a binary value in a binary positioning dataset differ from any others binary datasets at the same loci, we consider it as an inconsistent data, and refer to corresponding loci as the dynamic nucleosome-occupied domain (the dynamic nucleosome for short). The logic XOR was used to evaluate the differences between these datasets. We finally integrated those XOR results by using the arithmetic mean (see Methods, Figure 3). Correspondingly, we referred to this integrated data as the dynamic nucleosome profile. Interestingly, similar to the observation regarding the stable nucleosome profile, we found that the integrated map of the dynamic nucleosome profiles is well organised around the TSS (Figure 4C).

Recently, a review article has suggested that there is an approximately Gaussian (normal) distribution of nucleosome positions around particular genomic coordinates at most loci [22]. Intriguingly, as shown in Figure 4, we found that the average distance between these peaks is 166 bp in the stable distribution domain (Figure 4A), and 165 bp in the dynamic distribution domain (Figure 4C). These distances are consistent with the previously established uniform ~165 bp spacing of nucleosomes (including ~18 bp linker DNA) near the 5' end of genes [5], demonstrating that the organisation of chromatin in promoter regions is a universal mechanism, independent of interstrains differences in yeast.

To further observe the characteristics of nucleosome distributions, we examined two distinct nucleosome distributions near the ends of genes, where nucleosomes are generally considered to be fuzzy [5]. At the 3' end of the ORF, the NFRs surrounding the transcription termination sites (TTSs) are mainly formed by stable profiles (Figure 4B), whereas dynamic nucleosome occupancy rapidly decreases downstream of the TTS (Figure 4D). The stable and dynamic nucleosomes clearly differ in their distributions along the TTS, suggesting that the 3' end of the NFR may be formed mainly by the stable nucleosomes.

**Figure 5:**
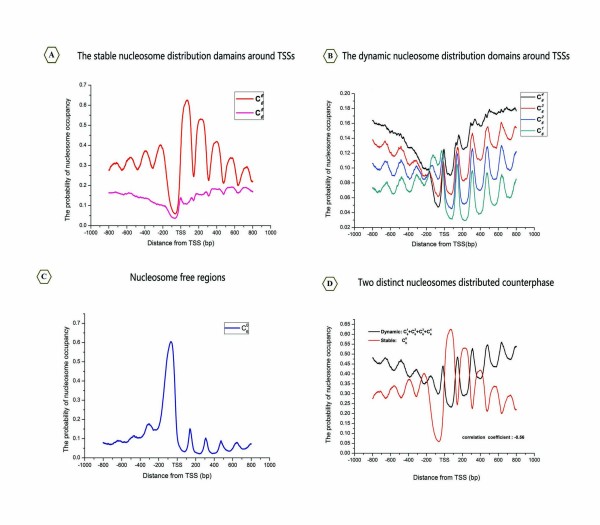
**Decomposition of the nucleosome distribution domains by the BDID model**. (A) The stable nucleosome probability distribution profiles, according to the binomial coefficients  and . (B) The dynamic nucleosome distribution profiles, including the curves of , ,  and . (C) The distribution of the NRF, profiles . (D) The counterphase between the two distinct nucleosome distributions. The dynamic nucleosome probability distribution curve (black) was integrated from , ,  and .

A possible concern is that the positions of the dynamic nucleosomes are almost exactly counterphase to the positions of the stable nucleosomes in promoter regions (Figure 4A, C), which may result from artefacts in our experimental method. In order to exclude the possibility that this phenomenon is caused by potentially undetected distributions, we checked whether another predominant nucleosome distribution pattern exists in the yeast genome. Naturally, we considered the six datasets as six independent and identically distributed (i.i.d) random samples. Based on statistical theory, we developed a binomial distribution induced decomposition (BDID) model to process the six position datasets and obtained the seven new reference maps according to the binomial coefficient  (see Methods). Surprisingly, the seven profiles, which represent the probability of nucleosome occupancy, can also be divided into two groups by comparing their phases with each other (Figure 5). The profiles  (Figure 5C), , ,  and  (Figure 5B, C) approximately belong to the dynamic domain, in which nucleosomes are either unmeasurable (NFRs) or destabilised. Alternatively,  and  (Figure 5A) may represent the stable domains, mainly formed by well-positioned nucleosomes. The stable domains and the dynamic domains are mapped in Figure 5D. Since the same tendencies were observed in the results from both the Logic Operation (Figure 4A, C) and the BDID model (Figure 5D), we concluded that the distributions we observed were unlikely to be artefacts of our experiment.

Two signal processing methods, namely the cross correlation and principle component analysis (PCA), were used to separately extract common and independent information from two occupancy datasets [3,4]. Interestingly, both signals also exhibit uniform phases and are distributed symmetrically around the TSS (Additional file 1: Supplemental Figure S2).

By pooling the nucleosome positioning information from six independent studies using the multi-angle analysis, our observations indicated that nucleosomes can be divided into two distinct classes: stable and dynamic. In contrast to simply analysing an individual experiment, it is statistically important to compile a compendium of six nucleosome positioning datasets based on an ensemble average. Our stable nucleosome map correlates well with the genome-wide distribution of well-positioned nucleosomes that had been previously defined [1,5,23]. Additionally, we observed that the distribution of dynamic nucleosomes mapped in our study correlates well with the fuzzy nucleosome score provided by Mavrich et al. [5]. However, we noted that the uniformity of both nucleosome positioning maps, with respect to the TSS, is much greater in our results than has been reported in any other studies (Figure 4A, C). Therefore, our results indicate that two distinct nucleosome maps can represent the characteristics of nucleosome distributions in vivo in a more refined manner than the stereotypical nucleosome maps derived from single experimental datasets. Indeed, the pervasive periodicity surrounding the TSS that we identified from the different data types further confirms that cross-platform information can faithfully reflect the robust mechanisms of nucleosome packaging in vivo.

### The measurable capacity of nucleosome positions in experiments

As previously described, nucleosomes can be divided into a stable group and a dynamic group. Obviously, the position of stable nuclesomes can be easily determined using various experimental methods. By contrast, the positions of dynamic nuclesomes are difficult to determine exactly through a single experiment. We separately used measurability and positioning signal-to-noise ratios (SNR) to assess the reproducibility and measurement accuracy of nucleosome location in promoter regions. The nucleosome measurability, which was defined as the average correlation coefficients between the six positioning datasets [2,3,5,6,11] around the TSS (-800 bp to +800 bp), quantifies the extent to which a set of independent experiments are able to measure the nucleosomes of each gene. In addition, we estimated the accuracy of nucleosomal positioning measurements by computing the SNRs, based on two distinct nucleosome distributions (see Methods).

**Figure 6:**
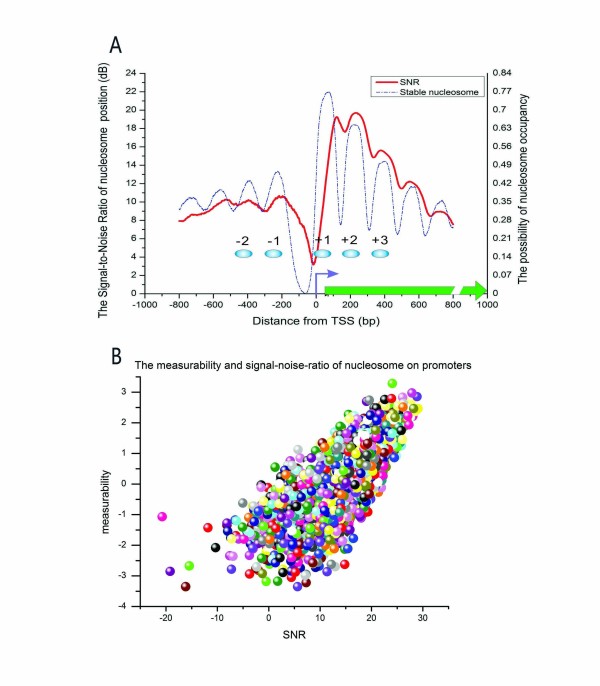
**The nucleosome positioning experimental SNRs and gene measurability**. (A) The red line is the experimental nucleosome SNR, calculated in a sliding window (200 bp) across promoter regions (surrounding TSS,-800 bp to 800 bp). The blue dotted line is the stable nucleosome distribution around the TSS. (B) Scatter plots for nucleosome SNR and measurability.

By aligning the SNR curve with the TSS, we observed that there were about nine nucleosomes with SNR values approaching or exceeding 10dB around the TSS (-800 bp to +800 bp). Notably, the SNR is lower at the TSS than in other areas, indicating that nucleosomes aggressively compete with transcription factors or RNA polymerase II at the TSS in vivo (Figure 6A).

Furthermore, in order to determine the relationship between nucleosome SNR and measurability, we generated a scatter plot of these two measurements for all genes (these measurements data assigned to each high-confidence transcript are available from Additional file 2). As expected, there was a positive correlation between SNR and measurability in promoter regions (correlation coefficient is 0.25, Figure 6B). These findings suggest that the regions in which the SNR value is near 10 dB contain nucleosomes that are easily and accurately detected using these experiments. In other words, stable nucleosome occupancy is more dominant than the dynamic nucleosome in these areas.

### Promoter nucleosome positioning patterns and regulatory properties: Dynamic nucleosomes do not serve as transcription barriers

It is generally accepted that nucleosomes decrease the accessibility of promoter elements, serve as an obstacle for transcription, and thus occlude the binding of transcription factors (TFs) to their binding sites [8]. Consequently, the emerging picture portrays nucleosomes as negative regulatory elements, and chromatin remodelling as the means to overcome repression by nucleosomes [23-27]. Several independent studies have also reported that gene expression activity correlates inversely with nucleosome occupancy in promoters: strongly expressed genes contain prominent NFRs, and genes that are expressed only at low levels tend to have promoters that are more readily occupied by nucleosomes [1,3,28]. Consistent with this, active promoters tend to exhibit reduced nucleosome occupancy, and gene activation is often associated with nucleosome destabilisation or eviction [3,26,29]. This view, however, may be an oversimplification, since many highly active genes are not depleted of nucleosomes [23].

**Figure 7:**
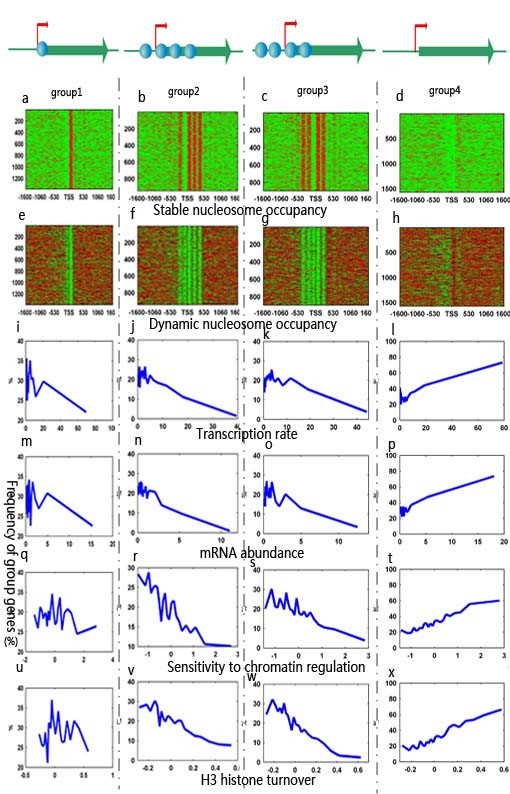
**Nucleosome position patterns and gene properties**. (a-h) k-means clustering for the set of 4,792 verified transcripts [30] with known TSS data, according to stable and dynamic nucleosome occupancy. Green represents areas depleted of nucleosomes, red areas are occupied. The frequency of each group of genes among 4,792 high-confidence transcripts with changing property values was calculated for each sliding 209 gene window. (i-x) The frequency of the four groups' genes were plotted as a function of various properties, including transcription rate, mRNA abundance, sensitivity to chromatin regulation and H3 turnover.

To address this problem, we investigated the relationship between nucleosomes and transcriptional properties using our cross-platform datasets. In our studies, we divided the 4,792 verified transcripts [5,30] into four significant groups by k-means clustering, based on the stable nucleosome map with a window of ~1600 bp surrounding the TSS (Figure 7a, b, c, d). We then examined whether the four groups of genes exhibited significant differences in terms of a variety of gene properties (namely transcription rate [12], mRNA abundance [13], sensitivity to chromatin regulation [14], and H3 histone turnover [15]). We first divided 4,792 high-confidence transcripts into 23 subsets of equal size (~209 genes each). Next, genes were sorted by corresponding property value, and 209 gene sliding windows were examined across each dataset. For each window, the percentage of genes in each group was plotted as a function of the window's average property value. Several interesting relationships are presented in Figure 7. Specifically, the curve significantly exceeding the genome-wide average of 20% within a group reflects a tendency for those genes to be significantly abundant in the corresponding regulation property value (Figure 7i, j, k, l, m, n, o, p, q, r, s, t, u, v, w, x).

As shown in Figure 7, the promoters without the stable +1 nucleosomes in group 4 exhibit higher values for gene properties than any of the others (Figure 7i, p, t, x). This finding is consistent with previous studies that have suggested a stable +1 nucleosome located on the TSS is critical for a gene's regulatory properties [5,31]. Furthermore, our studies also showed that the number of stable nucleosomes in coding regions significantly impacts on the activity of the corresponding group's genes. Specifically, the coding regions of highly expressed genes are significantly less likely to be occupied by stable nucleosomes than the coding regions of genes that are expressed at low levels or not at all. For instance, we observed that only those genes in group 1 with a stable +1 nucleosome show higher values of regulatory properties than the group 2 and group 3 genes (Figure 7i, m, q, u). Consistent with this, the number of stable nucleosomes in group 3 is lower than that of group 2, suggesting that the genes in group 3 are more active than those of group 2 (Figure 7j, k, n, o, r, s, v, w).

Second, we asked whether gene expression is always inversely correlated with nucleosome occupancy in promoter regions, as previous studies have reported [1,3,28]. To this end, we compared the nucleosome occupancy density of promoters in different groups based on the Lee et al. dataset [3]. Surprisingly, the group 4 genes exhibit the highest average nucleosome occupancy density in promoter regions, indicating that the dynamic nucleosomes and highly expressed genes are in concurrence. We also found that the nucleosome density distinctions are statistically significant (two sample t-test showed p < 3.47 × 10^-7 ^for all four groups). This observation is obviously in conflict with the conventional results that suggest gene expression always correlates inversely with nucleosome occupancy [1,3,28,32]. A possible explanation for the observed pattern of occupancy in our study is that the genes with high transcriptional activity require the formation of the disordered nucleosome, which may decrease residence time of the PolII during elongation, and may encourage transcription through the nucleosomal barriers.

**Figure 8:**
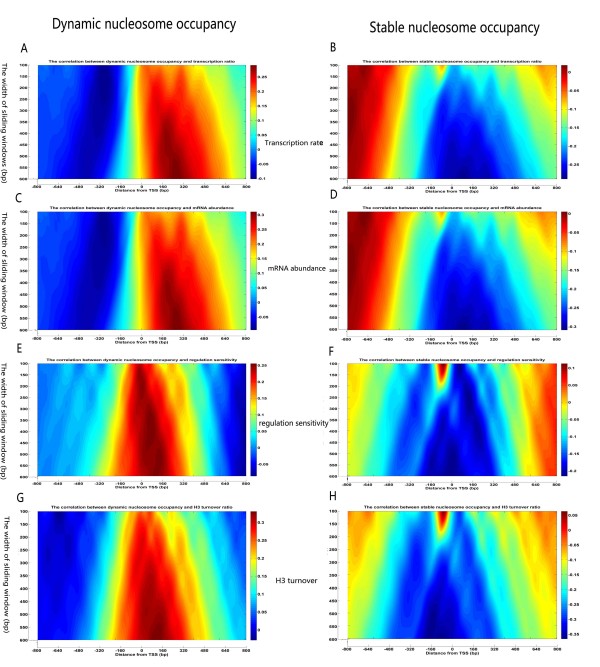
**Two distinct nucleosome local regulation correlation (LRC) results**. The distribution of the two classes of nucleosome LRC are mapped around the TSS (-800 bp to +800 bp). The width of the sliding windows ranges from 100 bp to 600 bp (1-bp steps), across both promoter and coding regions. (A), (C), (E), (G) The correlation between dynamic nucleosome LRC and corresponding gene properties. (B), (D), (F), (H) The correlation between stable nucleosome LRC and corresponding gene properties.

To further investigate whether there was a differential effect of the two distinct nucleosomes on gene transcription, we separately analysed the correlation between the two distinct nucleosome distributions and the regulatory properties in a genome-wide manner. Instead of the correlation between averaged nucleosome occupancy and transcriptional activity used in the previous studies [1,3,28], we employed a local regulatory correlation (LRC) method, which was defined as the correlation coefficients between the two classes of nucleosome densities in each window and the regulatory properties of high-confidence transcripts. We used a variable sliding window (ranged from 100 bp to 600 bp with a 1-bp step) across the promoter and parts of the coding regions for every gene (surrounding the TSS, -800 bp to +800 bp). Using a window, the local average nucleosome occupancy density was calculated. We then computed the Pearson correlation coefficients between the average occupancy densities and the corresponding gene properties, and plotted them as the heat maps (Figure 8).

As shown in Figure 8, we identified the stable and dynamic nucleosome's LRC regions for four gene properties. For transcription rate and mRNA abundance, the positive correlation scopes of the dynamic nucleosomes are similar and range from ~0 bp to ~+640 bp (Figure 8A, C). On the other hand, the stable nucleosomes show a negative correlation for the same LRC regions in terms of transcription rate and mRNA abundance (~-240 bp to ~+560 bp) (Figure 8B, D). Remarkably, we observed a weaker positive LRC region covering the upstream of TSS (~-190 bp to ~-30 bp) (Figure 8A, C), suggesting that the dynamic nucleosomes do not occlude the binding of TFs to regulatory elements in vivo. In contrast, the high intensity negative LRC regions in Figure 8B, D cover the promoter regions and part of the coding region (~-240 bp to ~+540 bp), demonstrating that the stable nucleosomes do serve as an obstacle for transcription. In accordance with our previous conclusion, the dynamic nucleosomes positively associate with transcriptional activity in coding regions, and do not serve as transcription barriers.

Regulation sensitivity, which was defined based on a smaller dataset compiled by Steinfeld et al[14], quantifies the extent to which the expression of each gene depends on the activity of chromatin regulators. Interestingly, we observed that the positive LRC regions of the dynamic nucleosomes (~-210 bp to ~+400 bp) are similar for both regulation sensitivity and H3 turnover [15](Figure 8E, G). These regions are also within the negative scopes of the stable nucleosomes (~-480 bp to ~+400 bp) (Figure 8F, H). Similar to regulation sensitivity, our results suggested that histone H3 turnover may also depend on the activity of chromatin regulators and may be accompanied by the presence of dynamic nucleosomes.

Instead of using the stereotypical nucleosome occupancy patterns and the previously suggested global correlation, we separately applied k-means clustering and LRC techniques to explore in detail the relationship between two distinct types of nucleosomes and gene regulatory properties. We determined that the dynamic nucleosomes positively correlate with gene properties, rather than serving as transcription barriers, a fact that has not been previously reported. In addition, we also found that the LRC maps for the two distinct types of nucleosomes may array alternately, keeping the phases spatially complementary (Figure 8).

### Insights into the connection between the stable +1 nucleosome position and the TSS

**Figure 9:**
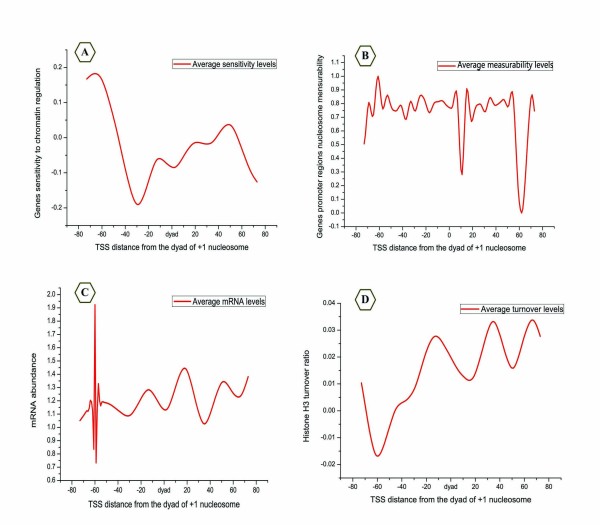
**The regulatory properties and the distance between the TSS and the dyad of the +1 nucleosome**. The averages of the properties of genes were plotted as a function of the distance between the dyad of the +1 nucleosome and the TSS. (A) Regulation sensitivity. (B) Nucleosome measurability. (C) mRNA abundance. (D) Histone H3 turnover.

Recent studies have shown that the exact position of the +1 nucleosome significantly impacts gene regulation [5,11,31]. In addition, independent experiments have shown that there exists a genome-wide bias for the TSS location at a site ~13 bp inside the upstream border of the +1 nucleosome, indicating that the +1 nucleosome is very specifically positioned [3,7,8]. However, less is known about the functional consequences of this connection. To address this problem, we examined whether the distance between the dyad of the +1 nucleosome and the TSS is crucial for the gene regulatory program. To this end, the average property of all genes was plotted as a function of the distance between the dyad of the +1 nucleosome and the TSS. Several notable relationships are presented in Figure 9, including the sensitivity to chromatin regulation [14], nucleosome measurability, mRNA abundance [13] and histone H3 turnover [15].

We observed a sharp change in the four signals when the TSS is located ~-60 bp from the dyad of the +1 nucleosome (Figure 9), a finding that has not been previously reported. Interestingly, with the exception of H3 turnover, all of the properties present maximum signal peaks at ~-60 bp from the dyad of the +1 nucleosome (~13 bp from the upstream border of the +1 nucleosome) (Figure 9A, B, C). The H3 turnover signal intensity drops sharply at this point (Figure 9D), suggesting that the nucleosomes in these special promoters are more stable than those found elsewhere. A possible explanation for the observed phenomenon is that the promoters in which the TSS is located ~-60 bp from the dyad of the +1 nucleosome may take advantage of a regulation program given an optimising chromatin context. Indeed, our results suggested that a site ~13 bp inside the upstream border of the +1 nucleosome is a sensitive regulation point in the yeast genome.

### The +1 nucleosome positions differ in TATA-containing and TATA-free promoters

We asked whether the promoters that display a unique structure are distinct in stable or dynamic nucleosome occupancy from those promoters without such a structure. To this end, we compared two sets of genes, defined in terms of functional and regulatory properties: TATA-containing and TATA-free genes [16]. We aligned and averaged the stable profile with respect to the TSS (-800 bp to +800 bp) for both TATA-containing and TATA-free genes.

**Figure 10:**
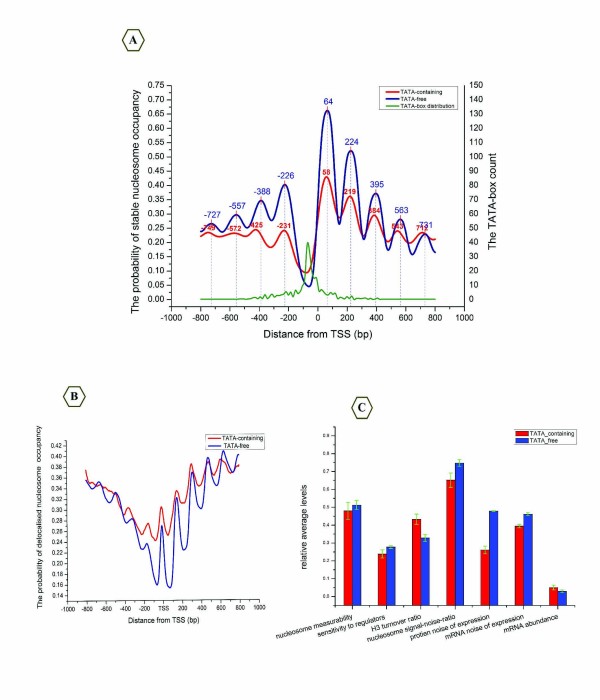
**Two distinct nucleosome distributions and the TATA-box**. (A) The stable nucleosome distribution based on TATA-containing (red) and TATA-free genes (blue). The green line is the TATA-box distribution in promoter regions. The digital data represent the coordinates of peaks relative to the TSS. (B) The dynamic nucleosome distribution based on TATA-containing (red) and TATA-free genes (blue). (C) Average values of properties that quantify the levels and variability of gene expression, nucleosome measurability and H3 histone turnover, based on TATA-contain genes (red) and TATA-free genes (blue). Error bars were calculated by bootstrapping.

Interestingly, the average distance between the dyad of the +1 nucleosome and the TSS is 58 bp on TATA-containing promoters, whereas this distance is 64 bp on TATA-free promoters. The difference of ~6 bp embedding the +1 nucleosome border indicates that TATA-containing genes require more protection than TATA-free genes. Notably, the signal intensity for stable nucleosomes on the TATA-containing genes is weaker than on the TATA-free genes (Figure 10A), confirming that most of the TATA-containing genes are strongly expressed and tend to be regulated by chromatin architecture [16,33]. Furthermore, the occupancies of dynamic nucleosomes on the TATA-containing genes are also higher than on the TATA-free genes when the dynamic profile is aligned with the TSS (-800 bp to +800 bp) (Figure 10B), suggesting that the TATA-containing genes consist mostly of in vivo active genes. Consistent with this conclusion, previous studies have reported that the TATA-containing genes are dedicated to a variety of stress responses and are highly regulated by a variety of chromatin modifications [16,26,33].

To further explore the connection between the TATA box and gene regulation, we separately analysed promoters that either contained or lacked a TATA-consensus sequence, according to the regulatory properties. We averaged the values of properties that quantify the relative variability of mRNA abundance [13], turnover of H3 histone promoter [15], sensitivity to regulators [14], expression noise [34], nucleosome measurability, and nucleosome SNR. In so doing, we observed a significant difference between the two distinct groups of genes (Figure 10C). Our results suggested that the distribution of the two classes of nucleosomes reflects complementary properties which not only affect chromatin structure, but also maintain the differences between TATA-containing and TATA-free promoters.

### Positioned-nucleosomes sliding in response to environmental transitions

Previous studies on the human [35], drosophila [36] and the yeast nucleosomes [20,21]suggested that the histone population changes in vivo depending on what that piece of chromatin is involved with at different developmental, physiological and disease stages. On the other hand, nucleosome positioning required for maintenance of the optimal internal milieu in one environment may be far from optimal in a different environment. Thus, when environmental conditions change abruptly, the cell must rapidly adjust its positioned-nucleosomes to adapt to the new conditions [37]. This means that those have been considered as static or well-positioned nucleosomes could become dynamic. However, it is not clear in what manner and to what extent the genome governs the movement of individual nucleosomes under environmental changes.

A series of conserved nucleosome positioning peaks around TSSs we observed in our reference data may indicate that there are some stable equilibrium points corresponding to the stable nucleosomes in the yeast genome (Figure 4A). It is possible that nucleosomes could be moved toward or far from these points to cope with specific environmental stresses. In order to examine this hypothesis, we first assessed positioned-nucleosome sliding level using two nucleosome positioning datasets which were collected before and after subjecting cells to heat shock [6]. Here, all nucleosomes were considered as the particles, and their positions were represented by their dyads. We searched for nucleosomes one-by-one along upstream and downstream of the TSSs in both datasets, regardless of their remodeling manners and distances from TSSs. Positioned-nucleosomes surrounding TSS were chosen because they have been well-characterized across a broad range of species and are amongst the most studied regions of the yeast genome.

**Figure 11:**
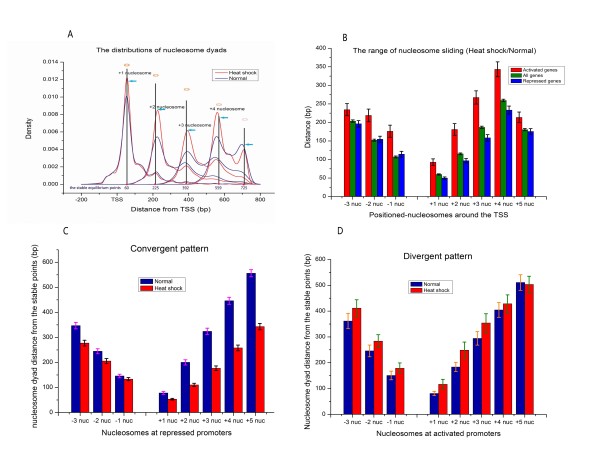
**The effect of heat shock on nucleosome positioning**. (A) Kernel density plot showing the distributions of +1,+2,+3, and +4 nucleosome dyads at the downstream of TSS before and after heat shock. Red lines show the centre of nucleosome distributions in heat shocked cells, and blue lines show that of in normally growing cells, respectively. The arrows indicate the distribution changes after heat shock at the stable equilibrium points. (B) Bar graph depicting the average sliding distances of positioned-nucleosomes after heat shock. Genes are divided into two groups according to their responding to environmental stress. Red bar presents nucleosome sliding range at the activated promoters, green bars show nucleosome sliding range at all promoters, and blue bars show nucleosome sliding range at the repressed promoters [38]. (C), (D) The average distances between the nucleosome dyads and correspondingly stable equilibrium points at the two classes of promoters before and after heat shock. Blue bar indicates nucleosomes in normally growing cells, and red bar indicates nucleosomes under heat shock. All error bars were calculated by bootstrapping. (The stable equilibrium points : -559, -393, -228, +60, +225, +392, +559 and +725 bp relative to TSS).

A kernel density plot was presented to show the changes in the nucleosome distribution for coding regions at verified transcriptions (Figure 11A)[30]. We noticed that four positioned-nucleosomes distributions displayed strengthened peaks after heat shock, suggesting that some nucleosomes were indeed shifted to those areas in response to the physiological perturbation. Strikingly, those new formed distribution peaks were also concentrated in the neighbourhoods of stable equilibrium points.

Since the kernel density evidently showed nucleosome sliding occurred during heat shock, it is necessary to further determine the scope of these displacements. To this end, we first classified promoters as two groups by their transcriptional activation under stress conditions, i.e. repressed and activated genes [38]. Based on data deriving from normally growing cells and heat-shocked cells, we calculated the average relative displacements for -3,-2,-1,+1,+2,+3,+4 and +5 nucleosomes. The results showed that nucleosome sliding exhibit broad dynamic range, especially those occupied at activated promoters and far from the TSS (range from 0 to ~350 bp))(Figure 11B). Our results are consistent with the previous observation that nucleosome remodeling is correlated with transcriptional activation [6].

In order to examine whether the sliding of individual nucleosomes is globally related to the conserved distribution domains during the physiological perturbation, we compared the average distances between eight positioned-nucleosomes and their corresponding stable equilibrium points before and after heat shock. The results showed that the patterns of nucleosome sliding can be mainly divided into two classes, i.e. convergent and divergent. In convergent pattern, the average distances in heat-shocked cells are shorter than that in normally growing cells, indicating that these nucleosomes tend to move towards their stable points (Figure 11C). In contrast, nucleosomes in divergent pattern are inclined to deviate from their stable points during heat shock (Figure 11D). Interestingly, we found that divergent pattern is mainly composed of activated promoters, whereas convergent pattern is mainly composed of repressed promoters (p < 10^-7^). Besides activated and repressed promoters, other genes globally exhibit a weak convergent pattern, suggesting that most of genes in yeast are basal expressions during transcriptional perturbation.

## Discussion

Recently, high-resolution tiling microarray and parallel DNA sequencing studies have yielded millions of measurements of nucleosomes [1-6,11]. Since yeast cultures are inherently similar in many respects, we would expect there to be nucleosomes at similar positions across different sample populations. In the previously published data, however, the derived nucleosome positions are inconsistent because biology is dynamic, making it difficult to determine the regulatory role of nucleosomes only based on an individual experimental data [1-8]. To address these problems, we developed several effective computational methods to mine nucleosome position characteristics across several published studies.

Our motivation for comparing six available experimental datasets was twofold. First, none of these independent studies was entirely consistent with any other according to the correlation coefficients between nucleosome position datasets (Figure 1 and 2), and we thus sought to use high-resolution maps to construct a set of new, high-confidence, reference maps. Our second motivation was to use these new reference maps to analyse the association between chromatin structure and gene regulatory properties.

Statistically, cross-platform information can more faithfully reflect the mechanism of nucleosome packaging in vivo than individual experiments. Through compiling genome-scale nucleosome positioning experimental data, we identified two nucleosome distribution maps that array alternatively and maintain mutually complementary phases in promoter regions. Indeed, our two classes show strong periodicity and central symmetry distribution around the TSS in the reference data, demonstrating that nucleosome organisation in the yeast genome is better conserved and more robust across two strains than has been reported in previous studies.

A growing number of studies are examining nucleosome destabilisation as an important mechanism in the epigenetic regulation of gene expression [5,6,8,39]. However, the mechanisms whereby nucleosome destabilisation-related processes affect regulatory properties are not well understood. It is therefore important to clarify whether the dynamic nucleosome distribution may be more relevant for transcriptional efficiency. To study this problem, we applied k-means clustering and the LRC method to systematically analyse the effects of the two classes of nucleosomes on the gene regulation program.

Several independent studies have reported that gene expression correlates inversely with nucleosome occupancy on promoters, and that active genes tend to exhibit reduced nucleosome occupancy [3,26,29]. However, in some cases, this assumption may be incorrect. When compared with the stable nucleosomes, our results suggest that the dynamic nucleosomes do not serve as negative regulatory elements in the genome (Figure 8). In fact, our studies provide statistical evidence that highly expressed genes are mainly occupied by the dynamic nucleosomes and have higher average nucleosome density than repressed genes (Figure 7). One possible explanation for the observed phenomenon is that RNA Pol II can only traverse the nucleosome under conditions in which at least one H2A/H2B dimer is lost [40], resulting in the nucleosomes being destabilised rather than evicted from these active genes. Consistent with this observation, a recent study also reported that a certain class of genes, the Occupied Proximal Nucleosome (OPN), displays a more evenly distributed and dynamic positioning of nucleosomes, with high occupancy close to the TSS [31]. Interestingly, the expression of these genes is also characterised by high transcriptional plasticity and sensitivity to chromatin regulation.

Recent studies have shown that the +1 nucleosome significantly impacts gene regulation [5,11,31] and the TSS tends to be ~13 bp inside the upstream border of the +1 nucleosome [1,2,5-7,29,41]. Using a statistically obtained, high-confidence, stable nucleosome map, we determined the exact distance between the dyad of the +1 nucleosome and the TSS for all genes. We then tested the hypothesis that the location ~13 bp inside the upstream border of the +1 nucleosome is an optimising chromatin conformation, which may achieve the maximum transcriptional outcomes while only requiring a minimal free energy cost for transcription elongation (Figure 9).

In addition, we established a connection between the stable +1 nucleosome and the TATA box. We found a subtle ~6 bp difference in the distance between the TSS and the dyad of the +1 nucleosome when comparing TATA-containing and TATA-free genes. Interestingly, the average length of the TATA box also ranges from 6 bp to 8 bp [16]. Thus, we suggest that there is a mechanism behind the above observation that may co-regulate gene expression. Alternatively, in order to shield TATA-containing genes from cryptic transcription, their TSSs may to be more closely embedded in the +1 nucleosome than TATA-free genes.

By carefully analysing the discrepancies between different studies, we found that uncertainty in nucleosome position is inherent in vivo. Further, we confirmed that even those well-positioned nucleosomes detected by previous studies could exhibit long-range sliding after heat shock. Therefore, it is worth noting that the nucleosomes that are considered to be static in our calculations could become dynamic under physiological conditions.

## Conclusions

By dividing nucleosomes into two classes according to their stable and dynamic characteristics, we considerably extend the characterisation of genome structure and gene architecture in yeast. Our genome-wide studies of the nucleosome position patterns and the LRC show that dynamic nucleosomes do not serve as transcription barriers. In fact, the dynamic nucleosomes are positively correlated with gene expression properties. Furthermore, we found that highly expressed genes contain significant dynamic nucleosome occupancy, whereas genes that are expressed at low levels tend to have promoters that are more readily occupied by stable nucleosomes.

We discovered that the distance between the +1 nucleosome and the TSS is as important as the nucleosome's specific position, suggesting that these genes have evolved an optimising chromatin context to respond to a regulatory program. On the other hand, we observed that the presence or absence of a stable +1 nucleosome in a promoter region can profoundly impact gene attributes. Furthermore, we found that the locations of stable +1 nucleosomes significantly differ in TATA-containing and TATA-free genes, suggesting that most of the TATA-containing genes are not only strongly expressed, but also tend to be protected by chromatin architecture.

By calculating the nucleosome measurability and the positioning SNRs, we estimated the reproducible capacity and measurement accuracy of nucleosomes in promoter regions genome-wide. According to positioning SNR, we determined that the areas surrounding the TSS (-800 bp to +800 bp) are critical for measuring and analysing nucleosome positions in practice.

Through comparing well-positioned nucleosome locations before and after heat shock, we found that the sliding range and moving patterns of nucleosomes are dependent on transcriptional activation, which further confirmed that nucleosomes are not static units because of biological dynamics.

## Methods

### Data restructuring and pre-processing

In order to process the data effectively, we used a binary sequence to reconstruct nucleosome position data. In our new dataset, the logical symbol 1 represents nucleosome packaging DNA, whereas symbol 0 represents linker DNA. Two data processing procedures are shown in Figure 3.

### Signal aligned with TSS

All signals were aligned with the TSS of 4,792 high-confidence transcripts derived from the literature [30] and defined in studies [3,5].

### Binomial distribution induced decomposition (BDID) model

We used a BDID model to process the six position datasets and obtain a new set of data. Here, the probability that a random variable *X(i) *with binomial distribution is equal to the value *k*, where *k *= 0, 1,...., *n*, is given by

The latter expression, , is known as the binomial coefficient, stated as "n choose k," or the number of possible ways to observe *k *"nucleosome appearing" from *n *experiments at the *i*_*th*_site. For example, the number of ways to observe 2 nucleosome appearances at the *i*_*th *_site in the six studies is "6 choose 2," or  = 15. Here, *P*{*X*(*i*) = *k*} is denoted as the probability of nucleosome appearance at the *i*_*th *_site along the chromatin. *p*(*i*) is the frequency of nucleosome appearance at the *i*_*th *_site, according to the six positioning datasets.

We denoted the binomial coefficients , , , , ,  and  as corresponding to the names of the probability profiles, which were calculated using the BDID model according to the k value (Figure 5). By comparing the phases of these curves with each other (Figure 5), we divided the seven curves into two categories. Specifically, the curves  correlate with NFRs, which exhibit areas of nucleosomal sparseness in the promoter regions (Figure 5C). The , ,  and  elements belong to the dynamic profiles, in which nucleosomes are characterised as either destabilised or fuzzy (Figure 5B). The profiles  and  represent the stable nucleosomes (Figure 5A).

### Nucleosome measurement SNRs

We proposed the following SNRs definition to handle the dynamic characteristic of nucleosomes in the experiments:

where S represents the average energy of the stable nucleosome signal and D represents the average energy of the dynamic nucleosome signal. Therefore, we referred to the SNR as the accuracy of the nucleosome positioning measurement.

## Authors' contributions

JF and XD designed the study, analysed the results and drafted the manuscript. JF also implemented the algorithms and carried out the experiments. XQ, CH, JW, JF and ZD contributed to the analysis and discussion. All authors read and approved the final paper.

## Supplementary Material

Additional file 1 **Data processing**. Two signal processing methods, namely logical operation, cross correlation and principle component analysis (PCA), were used to extract information from the two occupancy datasets [3,4]. Click here for file

Additional file 2 **Measurability and SNRs data**. The nucleosomal measurability and SNRs for the 4,792 high-confidence transcripts that were reported elsewhere [30]. Click here for file

## References

[B1] Yuan GC Liu YJ Dion MF Slack MD Wu LF Altschuler SJ Rando OJ Genome-scale identification of nucleosome positions in S. cerevisiae Science 2005 309 5734 626 630 10.1126/science.1112178 15961632

[B2] Albert I Mavrich TN Tomsho LP Qi J Zanton SJ Schuster SC Pugh BF Translational and rotational settings of H2A.Z nucleosomes across the Saccharomyces cerevisiae genome Nature 2007 446 7135 572 576 10.1038/nature05632 17392789

[B3] Lee W Tillo D Bray N Morse RH Davis RW Hughes TR Nislow C A high-resolution atlas of nucleosome occupancy in yeast Nat Genet 2007 39 10 1235 1244 10.1038/ng2117 17873876

[B4] Whitehouse I Rando OJ Delrow J Tsukiyama T Chromatin remodelling at promoters suppresses antisense transcription Nature 2007 450 7172 1031 1035 10.1038/nature06391 18075583

[B5] Mavrich TN Ioshikhes IP Venters BJ Jiang C Tomsho LP Qi J Schuster SC Albert I Pugh BF A barrier nucleosome model for statistical positioning of nucleosomes throughout the yeast genome Genome Res 2008 18 7 1073 1083 10.1101/gr.078261.108 18550805 PMC2493396

[B6] Shivaswamy S Bhinge A Zhao Y Jones S Hirst M Iyer VR Dynamic remodeling of individual nucleosomes across a eukaryotic genome in response to transcriptional perturbation PLoS Biol 2008 6 3 e65 10.1371/journal.pbio.0060065 18351804 PMC2267817

[B7] Ioshikhes IP Albert I Zanton SJ Pugh BF Nucleosome positions predicted through comparative genomics Nat Genet 2006 38 10 1210 1215 10.1038/ng1878 16964265

[B8] Segal E Fondufe-Mittendorf Y Chen LY Thastrom A Field Y Moore IK Wang JPZ Widom J A genomic code for nucleosome positioning Nature 2006 442 7104 772 778 10.1038/nature04979 16862119 PMC2623244

[B9] Peckham HE Thurman RE Fu Y Stamatoyannopoulos JA Noble WS Struhl K Weng Z Nucleosome positioning signals in genomic DNA Genome Res 2007 17 8 1170 1177 10.1101/gr.6101007 17620451 PMC1933512

[B10] Fernandez AG Anderson JN Nucleosome positioning determinants J Mol Biol 2007 371 3 649 668 10.1016/j.jmb.2007.05.090 17586522

[B11] Field Y Kaplan N Fondufe-Mittendorf Y Moore IK Sharon E Lubling Y Widom J Segal E Distinct modes of regulation by chromatin encoded through nucleosome positioning signals PLoS Comput Biol 2008 4 11 e1000216 10.1371/journal.pcbi.1000216 18989395 PMC2570626

[B12] Holstege FC Jennings EG Wyrick JJ Lee TI Hengartner CJ Green MR Golub TR Lander ES Young RA Dissecting the regulatory circuitry of a eukaryotic genome Cell 1998 95 5 717 728 10.1016/S0092-8674(00)81641-4 9845373

[B13] Beyer A Hollunder J Nasheuer HP Wilhelm T Post-transcriptional expression regulation in the yeast Saccharomyces cerevisiae on a genomic scale Mol Cell Proteomics 2004 3 11 1083 1092 10.1074/mcp.M400099-MCP200 15326222

[B14] Steinfeld I Shamir R Kupiec M A genome-wide analysis in Saccharomyces cerevisiae demonstrates the influence of chromatin modifiers on transcription Nat Genet 2007 39 3 303 309 10.1038/ng1965 17325681

[B15] Dion MF Kaplan T Kim M Buratowski S Friedman N Rando OJ Dynamics of replication-independent histone turnover in budding yeast Science 2007 315 5817 1405 1408 10.1126/science.1134053 17347438

[B16] Basehoar AD Zanton SJ Pugh BF Identification and distinct regulation of yeast TATA box-containing genes Cell 2004 116 5 699 709 10.1016/S0092-8674(04)00205-3 15006352

[B17] Wingert L Von Hippel PH The conformation dependent hydrolysis of DNA by micrococcal nuclease Biochim Biophys Acta 1968 157 1 114 126 4296058 10.1016/0005-2787(68)90270-0

[B18] McGhee JD Felsenfeld G Another potential artifact in the study of nucleosome phasing by chromatin digestion with micrococcal nuclease Cell 1983 32 4 1205 1215 10.1016/0092-8674(83)90303-3 6301684

[B19] Horz W Altenburger W Sequence specific cleavage of DNA by micrococcal nuclease Nucleic Acids Res 1981 9 12 2643 2658 10.1093/nar/9.12.2643 7279658 PMC326882

[B20] Guillemette B Bataille AR Gevry N Adam M Blanchette M Robert F Gaudreau L Variant histone H2A.Z is globally localized to the promoters of inactive yeast genes and regulates nucleosome positioning PLoS Biol 2005 3 12 e384 10.1371/journal.pbio.0030384 16248679 PMC1275524

[B21] Raisner RM Hartley PD Meneghini MD Bao MZ Liu CL Schreiber SL Rando OJ Madhani HD Histone variant H2A.Z marks the 5' ends of both active and inactive genes in euchromatin Cell 2005 123 2 233 248 10.1016/j.cell.2005.10.002 16239142 PMC2039754

[B22] Jiang C Pugh BF Nucleosome positioning and gene regulation: advances through genomics Nat Rev Genet 2009 10 3 161 172 10.1038/nrg2522 19204718 PMC4860946

[B23] Li B Carey M Workman JL The role of chromatin during transcription Cell 2007 128 4 707 719 10.1016/j.cell.2007.01.015 17320508

[B24] Kornberg RD Lorch Y Twenty-five years of the nucleosome, fundamental particle of the eukaryote chromosome Cell 1999 98 3 285 294 10.1016/S0092-8674(00)81958-3 10458604

[B25] Liu CL Kaplan T Kim M Buratowski S Schreiber SL Friedman N Rando OJ Single-nucleosome mapping of histone modifications in S. cerevisiae PLoS Biol 2005 3 10 e328 10.1371/journal.pbio.0030328 16122352 PMC1195719

[B26] Pokholok DK Harbison CT Levine S Cole M Hannett NM Lee TI Bell GW Walker K Rolfe PA Herbolsheimer E Genome-wide map of nucleosome acetylation and methylation in yeast Cell 2005 122 4 517 527 10.1016/j.cell.2005.06.026 16122420

[B27] Millar CB Grunstein M Genome-wide patterns of histone modifications in yeast Nat Rev Mol Cell Biol 2006 7 9 657 666 10.1038/nrm1986 16912715

[B28] Bernstein BE Liu CL Humphrey EL Perlstein EO Schreiber SL Global nucleosome occupancy in yeast Genome Biol 2004 5 9 R62 10.1186/gb-2004-5-9-r62 15345046 PMC522869

[B29] Lee CK Shibata Y Rao B Strahl BD Lieb JD Evidence for nucleosome depletion at active regulatory regions genome-wide Nat Genet 2004 36 8 900 905 10.1038/ng1400 15247917

[B30] David L Huber W Granovskaia M Toedling J Palm CJ Bofkin L Jones T Davis RW Steinmetz LM A high-resolution map of transcription in the yeast genome Proc Natl Acad Sci USA 2006 103 14 5320 5325 10.1073/pnas.0601091103 16569694 PMC1414796

[B31] Tirosh I Barkai N Two strategies for gene regulation by promoter nucleosomes Genome Res 2008 18 7 1084 1091 10.1101/gr.076059.108 18448704 PMC2493397

[B32] Liu X Lee CK Granek JA Clarke ND Lieb JD Whole-genome comparison of Leu3 binding in vitro and in vivo reveals the importance of nucleosome occupancy in target site selection Genome Res 2006 16 12 1517 1528 10.1101/gr.5655606 17053089 PMC1665635

[B33] Tirosh I Berman J Barkai N The pattern and evolution of yeast promoter bendability Trends in Genetics 2007 23 7 318 321 10.1016/j.tig.2007.03.015 17418911

[B34] Newman JR Ghaemmaghami S Ihmels J Breslow DK Noble M DeRisi JL Weissman JS Single-cell proteomic analysis of S. cerevisiae reveals the architecture of biological noise Nature 2006 441 7095 840 846 10.1038/nature04785 16699522

[B35] Okuwaki M Kato K Shimahara H Tate S Nagata K Assembly and disassembly of nucleosome core particles containing histone variants by human nucleosome assembly protein I Mol Cell Biol 2005 25 23 10639 10651 10.1128/MCB.25.23.10639-10651.2005 16287874 PMC1291234

[B36] Hamiche A Sandaltzopoulos R Gdula DA Wu C ATP-dependent histone octamer sliding mediated by the chromatin remodeling complex NURF Cell 1999 97 7 833 842 10.1016/S0092-8674(00)80796-5 10399912

[B37] Becker PB Nucleosome sliding: facts and fiction EMBO J 2002 21 18 4749 4753 10.1093/emboj/cdf486 12234915 PMC126283

[B38] Gasch AP Spellman PT Kao CM Carmel-Harel O Eisen MB Storz G Botstein D Brown PO Genomic expression programs in the response of yeast cells to environmental changes Mol Biol Cell 2000 11 12 4241 4257 11102521 10.1091/mbc.11.12.4241 PMC15070

[B39] Henikoff S Nucleosome destabilization in the epigenetic regulation of gene expression Nat Rev Genet 2008 9 1 15 26 10.1038/nrg2206 18059368

[B40] Kireeva ML Hancock B Cremona GH Walter W Studitsky VM Kashlev M Nature of the nucleosomal barrier to RNA polymerase II Mol Cell 2005 18 1 97 108 10.1016/j.molcel.2005.02.027 15808512

[B41] Sekinger EA Moqtaderi Z Struhl K Intrinsic histone-DNA interactions and low nucleosome density are important for preferential accessibility of promoter regions in yeast Mol Cell 2005 18 6 735 748 10.1016/j.molcel.2005.05.003 15949447

