# Supplementary material for: New insights into two distinct nucleosome distributions: comparison of cross-platform positioning datasets in the yeast genome
Source: BMC Genomics. 2010 Jan 15;11:33. doi: 10.1186/1471-2164-11-33 (PMC2824721; doi:10.1186/1471-2164-11-33)
Supplement: Additional file 1 — Data processing. Two signal processing methods, namely logical operation, cross correlation and principle component analysis (PCA), were used to extract information from the two occupancy datasets [3,4]. [file 1471-2164-11-33-S1.DOC]

Signal processing and analyzing

# The stable and dynamic nucleosome-occupied domians analyse:

Globally, when we aligned and averaged the AND data with respect to the TSSs in the genome-wide, we observed the stable nucleosome distributions continue at periodic intervals downstream of the TSS, with decreasing probabilities (or amplitudes) (Supplemental Figure 1 or Manuscript Figure 4 A). However, the average XOR data over high-confidence transcripts exhibits different occupancy properties. The dynamic nucleosome distributions also continue at periodic intervals downstream of the TSS, but their probabilities are increasing with distance from TSS (Supplemental Figure 1 or Manuscript Figure 4 C), suggesting that the density distribution of two groups nucleosomes exhibit different trends surrounding the TSS regions.

Locally, we can refer to the peaks of distribution profiles in both figures as the expected values of the nucleosomal dyads, but we can not ensure that the corresponding nucleosomes must be physically placed at the locations of these peaks, especially in a given gene.

Recently, a review article [4] has suggested that there may be an approximately Gaussian (or normal) distribution of nucleosome positions around particular genomic coordinates at most loci. Our results are consistent with their view. For example, in Supplemental Figure 1, every peak of profiles can be approximately considered as a nucleosome distribution center, and the scope of peak represents the standard deviation. According to our study, the average standard deviation in the stable nucleosome-occupied domains is greater than dynamic one. Essentially, the distances between peaks represent the standard deviation rather than the spaces of physically nucleosome occupancy. This phenomenon can be clearly observed at two neighboring areas A and B in Supplemental Figure 1.

## The nucleosome occupancy ratio data analyse

We asked whether occupancy ratios data have similar characteristics as positioning data analyzed in the text. In order to investigate the common characteristics between occupancy ratios data, we used the cross-correlation to find the common patterns in the Lee et al. and Whitehouse et al. studies [1, 2]. On the other hand, to discover the independent information between the two studies, we employed principal component analysis (PCA) to analyse them.

Our motivation for using two signal processing methods was twofold. First, in principle, the cross-correlation is a measure of similarity of two waveforms as a function of a time-lag applied to one of them. This is also known as a sliding dot product or inner-product. It is commonly used to search a long duration signal for a shorter, known feature. It also has applications in pattern recognition, single particle analysis, and cryptanalysis. Therefore, we use it to abstract the common information from the real number datasets, such as occupancy ratio data.

Second, PCA involves a mathematical procedure that transforms a number of possibly correlated variables into a smaller number of uncorrelated variables called principal components. The first principal component accounts for as much of the variability in the data as possible, and each succeeding component accounts for as much of the remaining variability as possible. Now it is mostly used as a tool in exploratory data analysis and for making predictive models**[3]**. Here, we used it to determine the independent information hidden in occupancy ratio datasets.

Interestingly, when we aligned and averaged the cross-correlation and PCA profile by TSS, both signals showed strong periodicity (Supplemental Figure 2A,B). Notably, when the distances between the peaks of two profiles were calculated, we found that the average space between these peaks is 160bp in the cross-correlation profile, and 163bp in the PCA profile respectively (Supplemental Figure 2C,D), which are similar to the results derived from the nucleosome positioning datasets(Manuscript Figure 4E,F).

# Figures

**
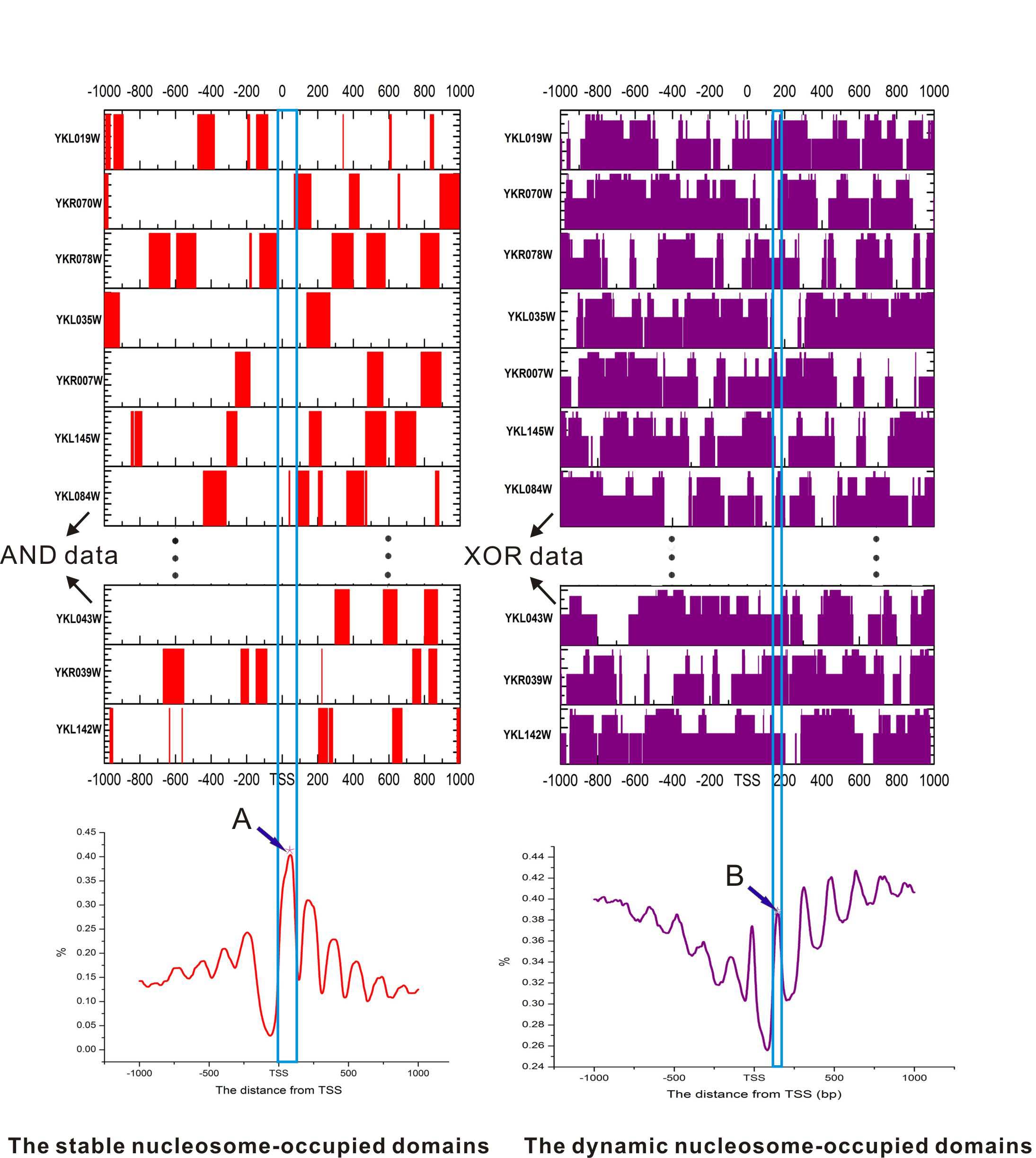
**

## Supplemental Figure 1 - **The program of obtaining the stable and dynamic nucleosome-occupied domians:**

the AND data and XOR data were aligned and averaged with respect to the TSSs of 4792 high-confidence transcripts (for space limited, only part of genes are illustrated in the graphs). Red graphs represent the stable nucleosome distributions. Purple graphs represent the dynamic nucleosome distributions. Areas of A and B represent two neighboring stable and dynamic nucleosome-occupied domains, respectively.


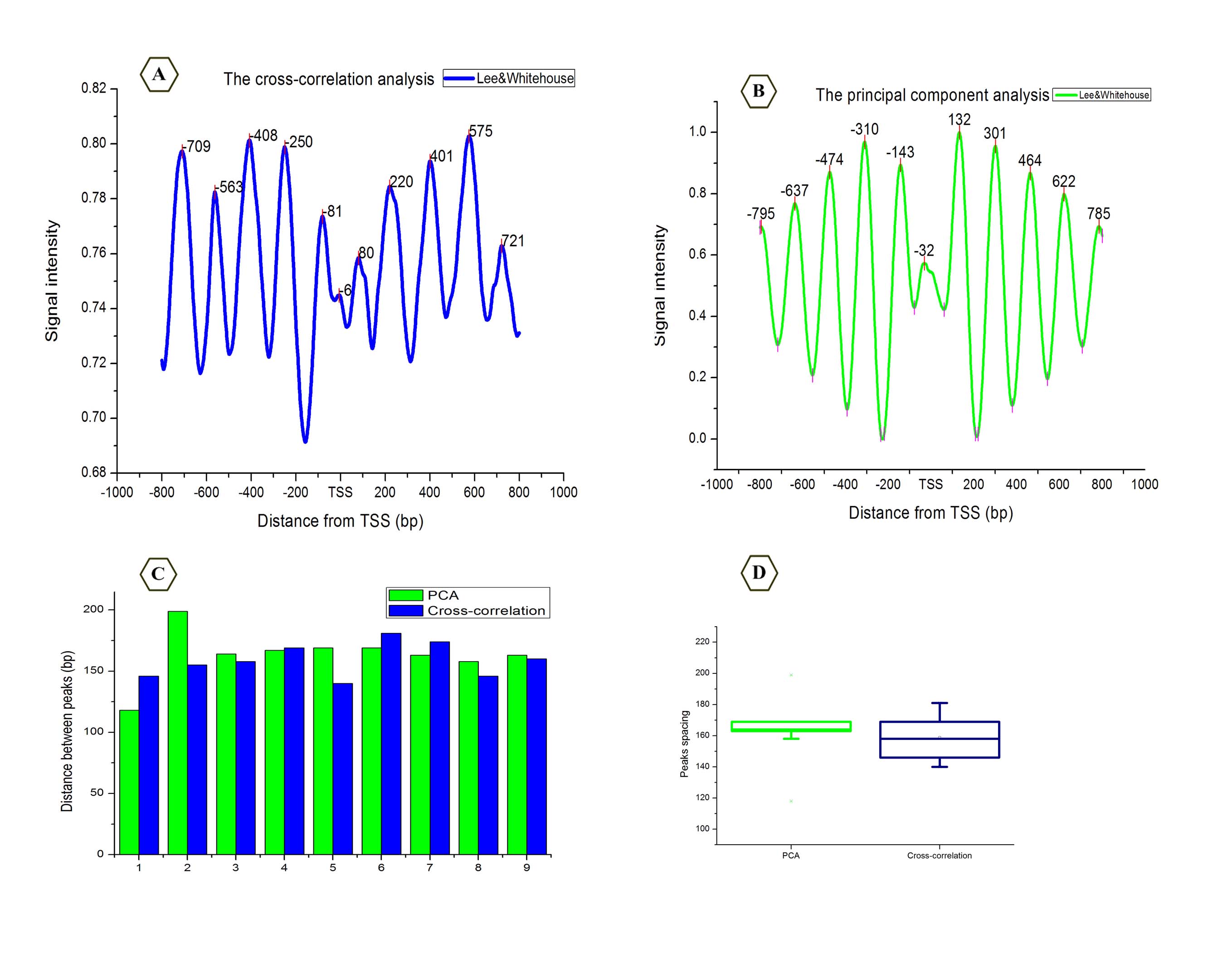


## Supplemental Figure 2 - Analyzing occupancy ratios data by cross-correlation and principle component analysis (PCA)

(A) The cross-correlation curve between Lee et al.[1] and Whitehouse et al.[2]. The digital values represent the coordinates of peaks relative to TSS. (B) The PCA curve between Lee et al. and Whitehouse et al. (C) The distances between peaks. Blue bars are the span of cross-correlation curve peaks, whereas green bars are the span of PCA curve peaks. (F) The box plot of average distance of peaks. Green box represents PCA curve, and blue box represents the cross-correlation curve.

# References

1. Lee W, Tillo D, Bray N, Morse RH, Davis RW, Hughes TR, Nislow C: **A high-resolution atlas of nucleosome occupancy in yeast**. *Nat Genet* 2007, **39**(10):1235-1244.

2. Whitehouse I, Rando OJ, Delrow J, Tsukiyama T: **Chromatin remodelling at promoters suppresses antisense transcription**. *Nature* 2007, **450**(7172):1031-1035.

3. Ringner M: **What is principal component analysis?** *Nature Biotechnology* 2008, **26**(3):303-304.

4. Jiang C, Pugh BF: **Nucleosome positioning and gene regulation: advances through genomics**. *Nat Rev Genet* 2009, **10**(3):161-172.
